# Acts and procedures concerning procedure-related deaths in South Africa 

**DOI:** 10.4102/phcfm.v5i1.453

**Published:** 2013-06-25

**Authors:** Stefan Jansen van Vuuren

**Affiliations:** 1 Department of Forensic Medicine, University of the Free State, South Africa

## Abstract

**Background:**

The act regarding procedure-related deaths in South Africa has changed recently (in 2007) and comprises a more encompassing description of possible procedure-related deaths. It subsequently includes unnatural deaths in cases where the patient died during a procedure, or as a result of a procedure, or where it can be shown that any aspect of such a procedure has been a contributory cause. The act does not qualify the ‘procedure’ and by definition includes all procedures, including anaesthesia.

**Objective:**

The objective of this article is to bring awareness to general practitioners regarding the legal requirements when dealing with suspected procedure-related deaths, and to outline some of the regulations pertaining to the management of such incidents.

**Methods:**

A thorough study, interpretation and clarification of the new legislature on procedure-related deaths were performed.

**Discussion:**

The onus of deciding which procedure-related deaths are unnatural has been removed from the doctor as the new act includes all such deaths. Certain aspects of the acts remain difficult to interpret and consultation with the appointed forensic pathologist in your area is still essential in all cases of a suspected procedure-related death. Healthcare workers should acquaint themselves with all these regulations and acts that are readily available in the Government Gazette.

## Abstrait

Les actes et les procédures concernant les décès liés à l’intervention en Afrique du Sud

**Présentation:**

La loi sud-africaine portant sur les décès associés à des actes médicaux a récemment été modfiée (en 2007) et inclut une description plus globale des décès qui pourraient être associés à un acte médical. Elle inclut ensuite les décès non naturels dans les cas où le patient est mort au cours d’un acte médical ou des suites d’un acte médical, où lorsqu’il est possible de montrer qu’un aspect d’un tel acte médical a contribué au décès du patient. La loi ne qualifie pas l’ « acte » et, par définition, inclut tous les actes médicaux, y compris les anesthésies.

**Objectif:**

L’objectif de cet article est de sensibiliser les médecins généralistes aux prescriptions légales en cas de décès suspectés d’être associés à un acte médical, et de souligner certaines réglementations relatives à la gestion de tels incidents.

**Méthode:**

Une étude détaillée de la nouvelle loi sur les décès associés aux actes médicaux a été réalisée, et son interprétation et sa clarification ont été entreprises.

**Discussion:**

Il n’incombe plus au médecin de décider quels décès associés à des actes médicaux sont non naturels, la nouvelle loi incluant tous ces décès. Certains aspects de la loi restent difficiles à interpréter et il est essentiel de consulter le médecin légiste assigné à votre secteur dans tous les cas de décès susceptible d’être associés à un acte médical. Les travailleurs de la santé devraient se familiariser avec ces réglementations et lois qu’ils pourront aisément trouver dans le Journal officiel du gouvernement.

## Introduction

Very few doctors know or understand the acts and regulations governing their profession, having had minimum exposure to them during undergraduate training. It is especially true concerning the acts governing the inquest (medico-legal examination or investigation) of an anaesthetic or procedure-related death. This document will outline current acts and regulations concerning such deaths, especially with regard to the duty of the doctor. Procedure-related deaths are a very important topic and this document will also provide guidance for the management of suspected procedure-related deaths.

The *Births and Deaths Registration Act* (Act 51 of 1992)^1^ specifically burdens the doctor to make a decision whether a death was natural or unnatural. However, it can sometimes be very difficult to ascertain whether a particular death is to be considered natural or unnatural, or if an inquest will be needed. A prominent factor might be a lack of confidence amongst doctors to make such a big decision. Furthermore, the curriculum for training of undergraduate medical students falls short in educating them in medical law and the application thereof.

Although most doctors and other healthcare workers (for the sake of this document ‘doctors’ will henceforth refer to all healthcare workers) are acutely aware of the rights of patients, but the specific acts and regulations concerning these rights are not always well-known, or there might be insufficient understanding of the implementation thereof. Patients have specific rights concerning their relationship with a doctor. Regulatory boards, such as the Health Professions Council of South Africa (HPCSA), exist to protect these rights, and to assist where they might be violated. The existing structures in the justice system (e.g. the Criminal Procedure Act) that protect ‘normal’ human rights are also applicable in the doctor-patient relationship. Subsequently it should not be possible for a doctor to murder someone under the pretense of doing a procedure on them. These laws and structures also protect patients against incompetent doctors. In South Africa, patients’ rights are protected by various laws and regulations, such as the *Health Professions Act*,^2^ the *Health Professions Amendment Act*^3^ and the *National Health Act*,^4^ as well as the Constitution. These acts ‘guarantee’ ethical and competent management of patients by their doctors.

The spectrum of patients consulting doctors varies from those with minor illnesses to some having life-threatening problems that require immediate intervention. The competent management of patients often requires certain procedures to be performed on them. Some of these procedures in themselves carry an inherent risk of morbidity and mortality, and patients can die during or as a result of a specific procedure. The abovementioned laws and regulations require certain measures to be taken when any procedure is to be performed on any patient. In the event of a death during or as a result of such a procedure, careful consideration regarding the circumstances of the death should be made to ensure the death was not caused by an act of commission or omission by the doctor(s) involved. The abovementioned acts and regulations, as well as other national documents (to be discussed in this document) ensure proper investigation of such deaths.

## Discussion

### Medical legislation of deaths in South Africa 

There are several acts that govern the management of deaths in South Africa. It is necessary for all doctors to acquaint themselves with these acts. The relevant acts will be discussed in this document in order to highlight some of the important sections in them.

- The *Births and Deaths Registration Act* (Act 51 of 1992)^1^ requires that a medical practitioner must issue a notification of death (form *BI 1663*) stating the cause of death. The cause of death should be stated as being either natural (with a primary cause of death) or unnatural. Section 15 of the Act states that: 
- Where a medical practitioner is satisfied that the death of any person who was attended before his death by the medical practitioner was due to natural causes, he shall issue a prescribed certificate stating the cause of death. (Section 15[1]) 
-   A medical practitioner who did not attend any person before his death but after the death of the person, examined the corpse and is satisfied the death was due to natural causes, may issue a prescribed certificate to that effect. (Section 15[2]) 
- If a medical practitioner is of the opinion that the death was due to other than natural causes, he shall not issue a certificate mentioned in subsection (1) or (2) and shall inform a police officer as to his opinion in that regard. (Section 15[3])
- In cases of unnatural deaths, the *Inquests Act* (Act 58 of 1959)^5^ provides for a medico-legal investigation (an inquest) to be held, including a medico-legal post-mortem examination. Sections 2 and 3 of this act also relate to the duty (by ‘any person’) to report unnatural deaths and the investigation of circumstances surrounding certain deaths. Sections 2 and 3 of the Act state that: 
- Any person who has reason to believe that any other person has died and that the death was due to other than natural causes, shall as soon as possible report accordingly to a policeman, unless he has reason to believe that a report has been or will be made by another person. (Section 2[1]) 
- Subject to the provisions of any other law providing for an investigation of the circumstances of any death, any policeman who has reason to believe that any person has died and that such a person has died from other than natural causes, shall (Section 3[1]): 

(1) Investigate or cause to be investigated the circumstances of the death or alleged death (Section 3[1] [a]). (2) Report or cause to be reported the death or alleged death to the magistrate of the district concerned, or to a person designated by the magistrate. (Section 3[1] [b]) 

- If the body of the person who has allegedly died from other than natural causes is available, it shall be examined by the district surgeon or any other medical practitioner, who may, if he deems it necessary for the purpose of ascertaining with greater certainty the cause of death, make or cause to make an examination of any internal organ or any part or any contents of the body, or any other substance or thing. (Section 3[2])
- In terms of the *National Health Act* (Act 61 of 2003)^4^ the following shall be deemed to be deaths due to unnatural causes. These deaths are defined as follows in the regulations to the Act:
- Any death due to a physical or chemical influence, direct or indirect, and/or related complications (this would by definition include procedure- and anaesthetic-related deaths, as by nature they involve physical and chemical influences).
- Any death, including those deaths that would normally be considered to be death due to natural causes, which in the opinion of the medical practitioner, has been the result of an act of commission or omission which may be criminal in nature.
- Any procedure related (including anaesthesia-related) death as contemplated in section 48 of the *Health Professions Amendment Act* (Act 29 of 2007). 
- Where the death is sudden and unexpected, or unexplained, or where the cause of death is not apparent. 

 According to the Inquests Act^5^ the doctor (or any person, for example appointed relations officers in their specific institution) must report any suspected unnatural death to a policeman as soon as possible. The sections that prescribe which deaths should be regarded as unnatural (see the *National Health Act*)^4^ gives very broad inclusions and the opinion of the appointed forensic pathologist in your area should be obtained in all doubtful cases.

## Anaesthesia- versus procedure-related deaths

The following discussion will serve to highlight the recent changes in the acts pertaining to procedure-related deaths. It will also explain the act and its interpretation and endeavor to help the doctor make decisions when faced with a possible procedure-related death.

In 2007 the *Health Professions Amendment Act* (Act 29 of 2003, section 48) substituted anaesthesia-related deaths’ with the more encompassing ‘procedure-related deaths’. The purpose of the substitution was to broaden the act to include procedures that do not require anaesthesia.

Section 56 of the previous *Health Professions Act* (Act 56 of 1974)^2^ stated:

The death of a person whilst under the influence of a general anaesthetic or local anaesthetic, or of which the administration of an anaesthetic has been a contributory cause, shall not be deemed to be a death from natural causes as contemplated in the Inquests Act, 1959 (Act 58 of 1959), or the Births and Deaths Registration Act, 1992 (Act 51 of 1992).

This act also qualified anaesthetic to include general and local anaesthetic. It included unnatural deaths in cases where the patient died whilst under the influence of anaesthetic (with no specific time period placed on the inclusion), and/or it can be shown that the administration of an anaesthetic has been a contributory cause.

According to this section, anaesthetic deaths included patients dying during anaesthesia due to, amongst others, the effects of the drug, malfunctioning of machines or equipment and anaesthetic procedural errors. Also, where it could be shown that the anaesthetic was a contributory cause, no time period was applicable and it could theoretically be years after the procedure had been performed. By definition of this act, procedures that did not require anaesthesia were excluded. These deaths would have been included as ‘unnatural deaths’ if it could be shown that the procedure caused the death, that is a death caused ‘by a physical influence’ as stipulated in the *National Health Act*.^4^

In 2007, section 56 of 1974 was substituted by section 48 of the *Health Profession Amendment Act* (Act 29 of 2007)^3^:

‘The death of a person undergoing, or as a result of, procedure of a therapeutic, diagnostic or palliative nature, or of which any aspect of such a procedure has been a contributory cause, shall not be deemed to be a death from natural causes as contemplated in the Inquests Act, 1959 (Act 58 of 1959), or the Births and Deaths Registration Act, 1992 (Act 51 of 1992).

The substituted section includes unnatural deaths in cases where the patient died during a procedure, or the patient died as a result of a procedure, or it can be shown that any aspect of such a procedure has been a contributory cause. 

The substituted section does not qualify the specific procedure, but it does include procedures of a therapeutic, diagnostic or palliative nature. Since the substituted section does not qualify the procedure, it includes by definition all procedures. The procedure also includes anaesthetic procedures, including administration of drugs in local or general anaesthesia. The act does not exclude such procedures as taking blood pressure, putting up an intravenous access line, or even a trivial procedure such as taking a patient’s temperature. At first this might sound excessive, but it also has to be agreed that, however improbable, it is possible to cause a death due to a complication of these ‘trivial’ procedures. For example, the possibility exists that an intravenous drip may cause sepsis and the consequent death of the patient. The inclusion of all procedures in section 48 removes the responsibility of deciding which deaths qualify to be investigated from the doctor and places it on the inquest magistrate or appointed person (forensic pathologist). It enforces the fact that all deaths possibly due to, or as result of a procedure, should be investigated by an inquest (formal or informal). If a patient dies during a procedure, or as a result of a procedure, or if it can be shown that the procedure contributed to the death, an inquest should be held to ascertain the liability of the person who performed the procedure.

Some procedure-related deaths can occur during a procedure. These deaths usually include patients that are acutely compromised by underlying natural or unnatural pathology, but can also include deaths due to an act of commission or omission by the managing doctor. The *Health Profession Amendment Act* (or regulations in the National Code of Guidelines for Forensic Practice in South Africa)^6^ does not specify when the procedure starts and when it ends. In general, a surgical procedure starts when the anaesthetist starts the placement of anaesthetic paraphernalia and ends when the patient is transferred from the recovery room, but it is up to the inquest magistrate to decide the specific timing of the procedure according to evidence available. The death does not necessarily have to be due to the procedure, it could have been as a result of the underlying ‘natural’ condition for which the procedure was performed. Even when the mechanism of death in these conditions was from a natural cause, the act stipulates that, due to the timing of the death, e.g. during the procedure, it must be regarded as unnatural.

When a patient dies ‘as a result of a procedure’ there is a direct causal relationship between the procedure and death. The death could be due to a known complication of the procedure or due to negligence in the performance of the procedure. There is no timeline relationship between the time of death and the cause of death. An example would be someone who dies of sepsis after a cosmetic procedure. It would be for the inquest magistrate to decide if the death was the result of an act of commission or omission by the doctor or due to an acceptable complication, and thus whether prosecution is needed. 

The determination of whether a ‘procedure has been a contributory cause’ is not always easy. Again, there is no time limit to this stipulation of the act. If it could be shown that there was sufficient contribution of the procedure to the cause or mechanism of death, the death must be considered unnatural. An example would be someone who dies of heart failure after life-saving triple bypass heart surgery, where the procedure itself could have caused a sudden deterioration of the underlying heart pathology. Again, it would be for the inquest magistrate to decide if the death was the result of an act of commission or omission by the doctor or due to a complication, and thus whether prosecution is needed. It would also be advisable to contact the forensic pathologist in your area for guidance.

## The inquest

According to the *Inquests Act* (Act 58 of 1959), all procedure-related deaths shall be investigated and thus should accordingly be reported to a police officer.^5^ If there is any uncertainty whether a medico-legal examination should be carried out, it is advisable to contact the Department of Forensic Medicine in the specific area to discuss the circumstances of the death with the forensic pathologist.

The investigation includes the following aspects:

- The opening of a case file (docket) by the South African Police Service – usually by an investigating officer. This docket contains all relevant information, including forms (in the Department of Health Free State Province: *HJ 199* and *HL 12D*) completed by the doctor and relevant parties, as well as affidavits and statements.
- The transport of the deceased to the government mortuary for a complete medico-legal post-mortem examination by a forensic pathologist or an appointed medical officer.
- The evaluation of the available information by a magistrate to assess the liability, or not, of the parties involved.

The inquest is a legal hearing where the particular case is assessed by the inquest magistrate.^7^ It could be in the form of a formal hearing where a prosecutor and witnesses are involved, or in the form of an informal ‘paper inquest’ where no witnesses are called. The magistrate will decide, after evaluating all the available information, if a case should be referred for formal prosecution of the parties involved. It does not conclude guilt of the parties involved and only determines whether a specific case has merit for prosecution, i.e. that the death was caused by an act of commission or omission by the doctors involved.^7^

Prosecution of the parties involved will entail a legal hearing in a court of law with a presiding judge (in the high court) or magistrate. It can lead to either a guilty or not guilty verdict. In the case of a guilty verdict, the accused involved will be sentenced according to criminal law. In the case of a not guilty verdict, the doctor can still be sued for damages by the deceased’s relatives (civil law). Independent of the outcome of the case (in the criminal or civil law proceedings), the doctor could also face disciplinary hearings by the statutory body involved, such as the HPCSA.^7^

## The medico-legal investigation of possible procedure-related deaths

The regulations for a medico-legal investigation are stipulated in the *National Health Act* (Act 61 of 2003)^4^ and the National Code of Guidelines for Forensic Pathology Practice in South Africa.^6^ The National Code of Guidelines for Forensic Pathology Practice in South Africa (the National Code) is a national document that describes the standard operating procedures to be followed by the forensic services in South Africa. It provides guidelines not only for the general management of forensic services, but also for the management of specific deaths (such as procedure-related deaths) by forensic pathologists. Both the *National Health Act* and the National Code specify the operating procedures to follow in cases of procedure-related deaths. The following paragraphs give a broad overview of some of the points that are important to doctors (they are explained fully in the abovementioned documents available in the Government Gazette).^6^

The procedure of managing possible procedure-related deaths broadly entails the following:

- As stated in the above acts (see the *Inquests Act*, Section 2 [1])^5^ the initial process in all unnatural deaths (including procedure-related deaths) includes involvement of the South African Police Service^6,7^ by reporting such deaths to a policeman. The investigating officer (IO) will give further instructions on the procedures to follow. The IO will open a case docket and provide a case number for reference.
- If possible, the body should be left in place at the scene of death, for example the operating theater in the case of a death during a surgical procedure, until instructed otherwise by a police officer. It is advisable to enquire from the police officer to whom the death is reported, if the body should be left in place or if it could be moved. It is important to note that only the appointed person from the Forensic Pathology Services can remove a body from the scene of a suspected unnatural death.^6^ 
- All paraphernalia and instruments used during the procedure (including drugs used, anaesthetic machines, etc.) should be left in place for the scene investigation. This might be problematic or difficult in South Africa, but it is necessary to enable proper medico-legal examination of the death.^6^
- All documentation, including forms to be completed, must be made available as soon as possible. Usually the managing doctor(s)– the surgeon and anaesthetist – as well as other medical staff involved (e.g. the professional nurse) must complete these forms. All parties involved should also make detailed notes for later reference. Forms might vary in specific areas. In the Free State Department of Health it includes the *HJ 199* and *HL 12d* forms. The National Code is presently in the process of being updated at which stage these forms will most probably be nationally uniform.
- In terms of the *Inquests Act* (section 3),^5^ only persons authorised by a magistrate or by the medical practitioner performing the post-mortem examination, may attend a post-mortem examination. It also states that ‘it is not desirable that a medical practitioner should perform a medico-legal post-mortem examination on one of his own patients, or the patient of his assistant, partner or locum tenens’.
- According to the National Code,^6^ the reports of medico-legal post-mortems may not be made available to any party except in certain conditions stipulated in the code. If any person (including the managing doctor) wishes to obtain a copy of the report, they must contact the IO involved.
- If the IO or medical practitioner performing the post-mortem (the forensic pathologist or registrar) contacts you for further information, it is advisable to give your co-operation as far as possible and to ensure that all communications are in writing.
- The National Code^6^ also states that the investigation of procedure-related deaths should be managed in consultation with the most senior regional or provincial forensic pathologist. It also states that a complete post-mortem must be performed in all cases that include gross examination of all systems, special dissection techniques where applicable, and the judicious selection and collection of specimens deemed necessary for special laboratory investigation. The regulations also point out that the post-mortem must be carried out by the authorised provincially appointed person and that the post-mortem must be carried out as soon as reasonably possible.

## Recommendations

Healthcare professionals are strongly advised to familiarise themselves with all legislation and procedural requirements applicable to procedure-related deaths. 

## Conclusion

The acts and regulations pertaining to healthcare professionals are very daunting, but careful consideration of the acts makes the interpretation thereof easier. All the regulations and acts regarding healthcare professionals are readily available in the Government Gazette and doctors should acquaint themselves with its content. The act regarding procedure-related deaths changed recently (in 2007) and comprises a more encompassing description of possible procedure-related deaths. Certain deaths that might have been considered natural in the past must now be classified as unnatural. The onus of deciding which procedure-related deaths are unnatural has been removed from the managing doctor, as the new act includes all such deaths. Certain aspects of the acts are difficult to interpret and the consultation with the appointed forensic pathologist in your area is still essential in all cases of suspected procedure-related deaths. For most doctors, the prospect of a procedure-related death investigation (or inquest) is intimidating, but it remains a necessary part in ensuring a safe environment for the patient.
